# Performance of large virus removal filters during AAV processing: Influence of flux and process disruptions

**DOI:** 10.1002/btpr.70106

**Published:** 2026-01-16

**Authors:** Akshay S. Chaubal, Asingsa W. Arachchige, Annabelle J. Zahn, S. Ranil Wickramasinghe, Xianghong Qian, Andrew L. Zydney

**Affiliations:** ^1^ Department of Chemical Engineering Pennsylvania State University University Park Pennsylvania USA; ^2^ Department of Chemical Engineering University of Arkansas Fayetteville Arkansas USA; ^3^ Department of Biomedical Engineering University of Arkansas Fayetteville Arkansas USA

**Keywords:** adeno‐associated virus (AAV), downstream processing, internal polarization, process disruptions, viral clearance, virus filtration

## Abstract

As adeno‐associated viral vectors (AAV) continue to advance through the clinical pipeline, effective downstream purification strategies must be developed to ensure bulk drug purity and safety. AAV are produced within mammalian cells, bringing forth risks associated with viral contamination. Although existing downstream operations provide some degree of viral inactivation and removal, regulatory agencies have recommended the incorporation of a dedicated virus removal filtration step to ensure robust viral clearance. Recently published studies have demonstrated that membrane filters with nominal pore sizes between 35 and 50 nm can provide effective AAV transmission while removing larger viruses, although these results were obtained over a limited range of conditions. This study represents the first investigation into the effects of filtrate flux and process disruptions on virus reduction filtration for AAV. Experiments were performed using purified AAV capsids and carboxylate‐modified polymeric nanoparticles with a nominal diameter of 20 nm. Initial results confirmed that both systems exhibited nearly identical transient transmission profiles during virus filtration. Virus filtration performed at various filtrate fluxes (between 20 and 185 L/m^2^/h) revealed that moderately higher AAV yield may be obtained at lower fluxes. The data were analyzed using a modified internal polarization model, which was extended to account for the effects of process disruptions on transient particle transmission and recovery. Process disruptions were employed to increase AAV yield beyond 99% without compromising overall clearance of large viruses. At least a 4‐log reduction in xenotropic murine leukemia virus (XMuLV) was observed under all conditions tested, even following multiple process pauses.

## 1 INTRODUCTION

Adeno‐associated viral vectors (AAV) are at the forefront for delivery of gene therapies, largely due to their non‐pathogenic nature and broad tissue tropism.^1^ AAV are produced within mammalian host cells (most commonly HEK 293 cells), which are lysed to release the AAV capsids into the culture media. The ensuing downstream purification process traditionally involves clarification, capture chromatography, polishing, and then ultrafiltration/diafiltration to ensure effective removal of process‐related impurities and contaminants.^2^

One of the challenges in AAV purification is viral clearance, which is a major concern for all biotechnological products derived from mammalian cells,^3^ including genetically engineered viral vectors like AAV. Viruses can be endogenous to the mammalian cell line, or they can arise from adventitious introduction during manufacturing.^4^ A number of the unit operations designed for AAV purification inherently provide some degree of viral inactivation and removal. For example, detergents used during cell lysis can break down the outer membrane of enveloped retroviruses.^5^ The low‐pH elution used to recover AAV from the affinity step can also inactivate viral contaminants,^6^ although prolonged exposure to low pH often results in some degree of AAV degradation.^7^ Affinity/polishing chromatography can also provide significant virus removal during washing of the bound AAV capsids.^8^ As noted in Annex 6 of ICH Q5A, “viral clearance studies should be performed to determine virus reduction factors for the relevant step(s) of the production process”.^3^

Virus removal filtration is ubiquitous in the processing of smaller monoclonal antibody (mAb) therapeutics, with membranes having nominal pore sizes of ~20 nm providing at least a 4‐log reduction value (LRV) of parvoviruses along with essentially complete removal of larger retroviruses.^9^ However, as AAV are parvoviruses, these traditional virus removal filters would retain AAV capsids (which are ~25 nm in diameter). Some recent studies have demonstrated that virus filters with nominal pore sizes of 35–50 nm can provide effective AAV transmission while removing larger viruses.^10^, ^11^, ^12^ Hermens et al.^13^ showed that the Planova 35N membrane could effectively remove a rod‐shaped baculovirus used in the production of AAV in insect cells, although data were only reported at a single transmembrane pressure of 16 kPa. Long et al.^10^ showed that the Viresolve NFR membrane provided very high removal of XMuLV (80–100 nm) and pseudorabies virus (120–200 nm), with AAV recoveries of 86% and 95% in two separate experiments. Namila et al.^11^ provided a more extensive study using two commercial virus removal filters (labeled Filter A and Filter B); AAV yields ranged from 81% to 109% with high removal of both Adv‐5 (70–90 nm) and SV‐40 (40–50 nm) viruses. Although these studies provide an initial proof‐of‐concept (summarized in Table 1), results were obtained over very limited operating conditions with little mechanistic insights into the factors controlling the performance of these larger pore size virus removal filters.

**TABLE 1:** Summary of AAV virus filtration studies reported in the literature.

Virus filter chemistry	Filter pore size	Average pressure	AAV yield	Model virus; LRV	References
Cuprammonium‐regenerated cellulose	35 nm	16 kPa	94%–102%	Baculovirus; LRV ≥6.1	^13^
Cuprammonium‐regenerated cellulose	35 nm	83 kPa	81%–107%	Adv‐5; LRV ≥5.2 SV‐40; LRV ≥5.6	^11^
Polyvinylidene fluoride (PVDF)	50 nm	190 kPa	83%–109%	Not reported	^11^
Polyethersulfone (PES)	50 nm	207 kPa	86%–95%	XMuLV; LRV ≥6.0 PRV; LRV ≥5.4	^10^
Polyethersulfone (PES)	50 nm	Not reported	108%	Not reported	^12^

The objective of this study was to obtain a more detailed understanding of the behavior of the Planova™ 35N virus removal filter during the processing of AAV therapeutics. In particular, experiments were performed to evaluate the effect of filtrate flux and process disruptions (defined as a shutdown or significant reduction in flux/pressure during the filtration process) on both AAV yield and large viral clearance. These factors have previously been shown to significantly impact virus retention by parvovirus filters,^14^, ^15^, ^16^, ^17^, ^18^ but have never been studied with larger pore size filters required for AAV processing. Data were obtained with both purified AAV capsids and model fluorescent nanoparticles, with the latter having previously been used to probe virus capture in parvovirus removal filters.^19^, ^20^ Results were analyzed using a modified internal polarization model, which was developed by extending prior models that described the retention of small parvovirus during traditional virus filtration.^21^, ^22^

## 2 MATERIALS AND METHODS

### 2.1 AAV

AAV were produced within HEK 293 suspension cell culture as described in the literature.^23^, ^24^, ^25^, ^26^ Chemical lysis was performed using 10% (vol/vol) AAV‐MAX™ lysis buffer (ThermoFisher Scientific, Waltham, MA, USA) to release the AAV capsids. The resulting suspension was clarified using BioOptimal™ MF‐SL tangential flow microfilters provided by Asahi‐Kasei Life Science Corporation (Tokyo, Japan) as described elsewhere.^24^ The clarified cell lysate was then purified by affinity chromatography using POROS™ GoPure™ AAVX pre‐packed columns (ThermoFisher Scientific, Waltham MA, United States) connected to an ÄKTA™ Pure chromatography system. The final purified AAV product was suspended in either 20 mM Tris with 150 mM NaCl or 10 mM phosphate‐buffered saline, both at pH 7.4. The purified AAV2 feedstock contains some residual Pluronic F‐68 that was added during the initial production/harvesting process; additional Pluronic F‐68 was spiked into the purified AAV2 feed to a concentration of ~0.05% wt/vol to improve capsid stability. Limited experiments were also performed with an AAV2 feed obtained from an industrial collaborator.

AAV yield was measured using quantitative polymerase chain reaction (qPCR), which measures the number of viral genomes (vg) per unit volume. AAV transmission in grab samples obtained during the filtration process was quantified using UV absorbance at 280 nm, which was correlated to AAV concentration using calibration curves constructed with known standards.

### 2.2 Nanoparticles

The virus filters were also challenged with Fluospheres™, carboxylate‐modified polystyrene latex nanoparticles (ThermoFisher Scientific, Waltham, MA, USA) with nominal particle diameters of 20 nm (Catalog #: F8786), 40 nm (Catalog #: F8770), and 100 nm (Catalog #: F8801). The carboxylate modification gave the nanoparticles a net negative charge under aqueous conditions. Feed material for virus filtration was generated by diluting stock suspensions of the 20 nm nanoparticles into 10 mM phosphate‐buffered saline to a concentration of ~7 × 10^11^ particles/mL. The nanoparticles were then sonicated at 35 kHz for 10–15 min prior to use to break up any aggregates that may have formed during storage at 4°C. Nanoparticle concentrations were determined by fluorescence intensity using an Infinite m200 Pro microplate reader (Tecan, Mannedorf, Switzerland) using appropriate excitation and emission wavelengths.

### 2.3 Xenotropic murine leukemia virus (XMuLV)

Xenotropic murine leukemia virus (XMuLV) was propagated in mink lung host cells (ATCC® CCL‐64™) obtained from the American Type Culture Collection (ATCC). The cells were cultured in Dulbecco's Modified Eagle Medium (DMEM) with high glucose (Catalog #: D5796), supplemented with 1% sodium pyruvate and 10% fetal bovine serum (FBS). The cells were seeded in culture flasks at an initial density of 1.5 × 10^4^ cells/cm^2^ and maintained under standard cell culture conditions of 37°C and 5% CO₂. The XMuLV was harvested on days 4, 6, and 8, with the harvest fluid subsequently purified as described in the literature.^27^ Briefly, virus‐containing culture supernatant was first concentrated and washed with TNE buffer (10 mM Tris, 150 mM NaCl, and 1 mM EDTA) using Amicon Ultra‐15 Centrifugal Filters with 100 kDa regenerated cellulose membranes (MilliporeSigma, Burlington MA, USA). The virus was further purified via high‐speed ultracentrifugation (Beckman Coulter, Brea, CA, USA) to remove cell debris and remaining impurities while concentrating the XMuLV. The XMuLV pellet was resuspended in 2 mL of TNE buffer and filtered through a 0.2 μm syringe filter. The purified XMuLV stocks were stored at −80°C for long‐term use.

XMuLV titer was determined using the 50% tissue culture infectious dose (TCID_50_) method. The TCID_50_ assay was performed using PG‐4 (ATCC® CRL‐2032) cells seeded at 3150 cells‐per‐well into 96‐well plates, resulting in 10%–50% confluency after 24 h. The following day, XMuLV stock samples were diluted 10‐fold and added to each well, with 12 replicates per dilution. After 7 days of incubation, the infected cells were counted to determine the TCID_50_/mL value using the Spearman‐Kaber method at a 95% confidence level.^28^ When no virus particles in the filtrate samples were detected by TCID_50_, the more sensitive large volume plating (LVP) assay was used to determine the titer of XMuLV in the filtrate. More details of the LVP assay were described previously.^18^

### 2.4 Dynamic light scattering (DLS)

Dynamic light scattering (DLS) was used to evaluate the size distribution and zeta potential of the AAV and nanoparticle feedstocks. DLS data were obtained with a Zetasizer Nano ZS (Malvern Panalytical, Malvern, UK) instrument. For particle size analysis, 70 μL samples were loaded into a quartz cuvette, with measurements performed in triplicate over 20–100 repeat runs. Zeta potential was measured after appropriate sample dilution in 10 mM phosphate‐buffered saline at pH 7.4, with ~700 μL of material loaded into a folded capillary cell. All zeta potential measurements were performed in triplicate over 40 repeat runs. AAV samples were filtered through 0.2 μm sterilizing‐grade filters prior to DLS to remove any large aggregates.

### 2.5 Virus filtration

Virus filtration was performed at bench‐scale using Planova™ 35N hollow fiber modules having an effective membrane area of 10 cm^2^ provided by Asahi‐Kasei Life Science Corporation (Tokyo, Japan). The membranes are made from cuprammonium‐regenerated cellulose, with an average pore diameter of 35 ± 2 nm.^29^ Prior to each experiment, the module was flushed with 20 L/m^2^ of water‐for‐injection (WFI) through each of the three outlet ports (one retentate and two filtrate ports) to ensure complete wetting of the membrane and removal of any trapped air. The modules were then equilibrated with a surfactant‐free buffer that matched the buffer conditions of the feed. The normalized buffer permeability was evaluated from data for the filtrate flux at several transmembrane pressures; all membranes had permeabilities between 15 and 17 L/m^2^/h/psi.

Filtration experiments were performed in “dead‐end” (normal flow) mode under constant‐pressure or constant‐flux conditions, with the hollow‐fiber module mounted vertically as per the manufacturer's instructions. Constant‐pressure filtration utilized a compressed air source to pressurize the feed reservoir, whereas constant flux experiments used a peristaltic pump placed immediately upstream of the module to ensure a stable filtrate flux over time. Inline (grab) samples were collected during the experiment(s) to evaluate the particle/virus transmission:

(1) $$\text{Transmission}=\frac{C_{\text{filtrate}}}{C_{\text{feed}}}$$

where $C_{\text{filtrate}}$ and $C_{\text{feed}}$ are the concentrations of nanoparticles or AAV in the filtrate and feed samples, respectively. Clearance of large virus was evaluated from bulk samples taken of the initial feed and final filtrate reservoirs at the start and end of each experiment, with results presented in terms of the log‐reduction value (LRV):

(2) $$\mathrm{LRV}=-\log_{10}\left(\frac{C_{\text{filtrate}}}{C_{\text{feed}}}\;\right)$$

All Planova 35N membrane modules were treated as single‐use and discarded at the end of each run.

### 2.6 Confocal microscopy

Nanoparticle capture within fouled filters was visualized using confocal microscopy. Immediately after challenging the Planova 35N with the Fluospheres, the hollow fiber modules were dried by clamping the filtrate ports and allowing air to flow through the inner fiber lumens. The outer casing was cut open, individual fibers were removed, and the membranes were prepared for confocal imaging as described in the literature.^19^ In brief, fibers were cut into small pieces, embedded in resin, and then sectioned into thin slices using a Leica CM1950 Cryostat Microtome. The cut samples were placed on transparent slides, covered with a drop of Antifade Mountant (Molecular Probes, Inc., Eugene, OR, USA), and sealed with a cover glass.

Images of the fluorescent nanoparticles captured within the structure of the Planova 35N virus filters were taken using an Olympus Fluoview™ 1000 confocal scanning laser microscope. High‐intensity lasers were used to excite the nanoparticles, with the emitted fluorescent light collected through an oil objective lens. Quantitative profiles of particle capture vs. penetration depth were obtained using open‐source ImageJ image‐processing software (https://imagej.net/ij/).

## 3 RESULTS AND DISCUSSION

### 3.1 AAV/fluosphere characterization

**FIGURE 1:**
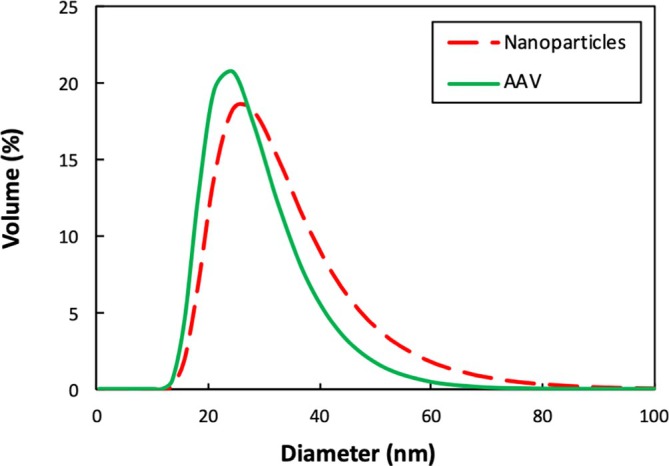
Particle size distributions of AAV and 20 nm Fluospheres measured via dynamic light scattering.

The size distribution of purified AAV and 20 nm Fluospheres were evaluated by dynamic light scattering (DLS) with results presented in Figure 1. The purified AAV capsids display a number‐averaged diameter of 23 nm, consistent with the expected size of monodisperse AAV as reported in the literature.^23^, ^26^, ^30^ The Fluospheres exhibited a number‐average diameter of 24 nm, with a similar but somewhat broader size distribution than that for the AAV.^31^ In particular, 21% of the Fluospheres had a size >35 nm (based on integration of the DLS peak) compared to only 10% of the AAV capsids. Additionally, both systems were negatively charged—the zeta potentials of the AAV and Fluospheres in 10 mM phosphate‐buffered saline at pH 7.4 were − 11 ± 1 mV and −35 ± 2 mV, respectively. These results confirm that the AAV and nanoparticles exhibit similar physical characteristics, suggesting that the nanoparticles may serve as an effective surrogate for studying the behavior of AAV during virus filtration. The use of model nanoparticles avoids the high batch‐to‐batch variability typically present within AAV feed material,^11^ which can make it difficult to interpret small differences in performance between experiments. Furthermore, nanoparticle fluorescence provides a rapid and low‐cost method for quantifying concentration and particle capture (by confocal microscopy). This facilitates mechanistic and reproducible virus filtration studies.

### 3.2 AAV/Fluosphere transmission

**FIGURE 2:**
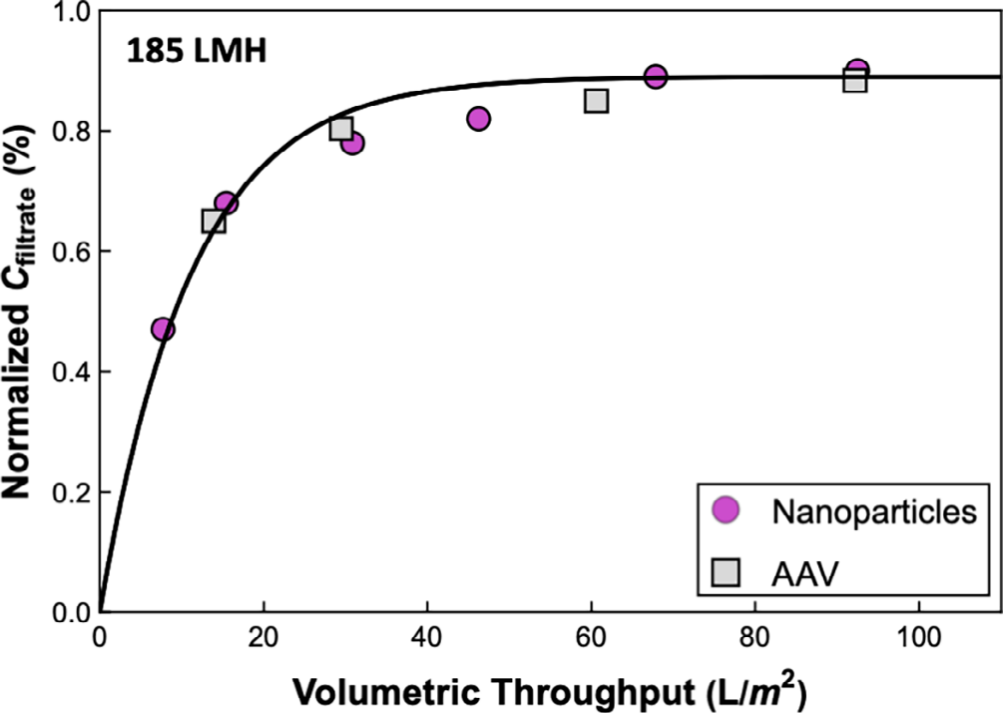
Normalized filtrate concentration (C_filtrate_/C_feed_) as a function of volumetric throughput during constant‐flux operation at 185 L/m^2^/h with AAV (squares) and 20 nm nanoparticles (circles) using a feed concentration of ~7 × 10^11^ particles/mL. Solid curve is model calculation using Equation (4) with x=0.89 and $S_{a}=9.0\times 10^{-4}$.

Figure 2 shows results for transmission of the AAV and 20 nm Fluospheres through the Planova 35N (in two separate experiments) as a function of the volumetric throughput, defined as the cumulative filtrate volume normalized by the membrane area. In both cases, ~100 L/m^2^ of a suspension with a particle concentration of ~7 × 10^11^ particles/mL were processed through the Planova 35N virus filter at a constant filtrate flux of 185 L/m^2^/h. Inline (grab) samples were collected to evaluate the transmission. The results for the AAV and Fluospheres are in excellent agreement, validating the use of the Fluospheres as a model for AAV. In both cases, the transmission increases from about 50% in the first sample (at V/A = 8 L/m^2^) to nearly 90% at high throughput.

**FIGURE 3:**
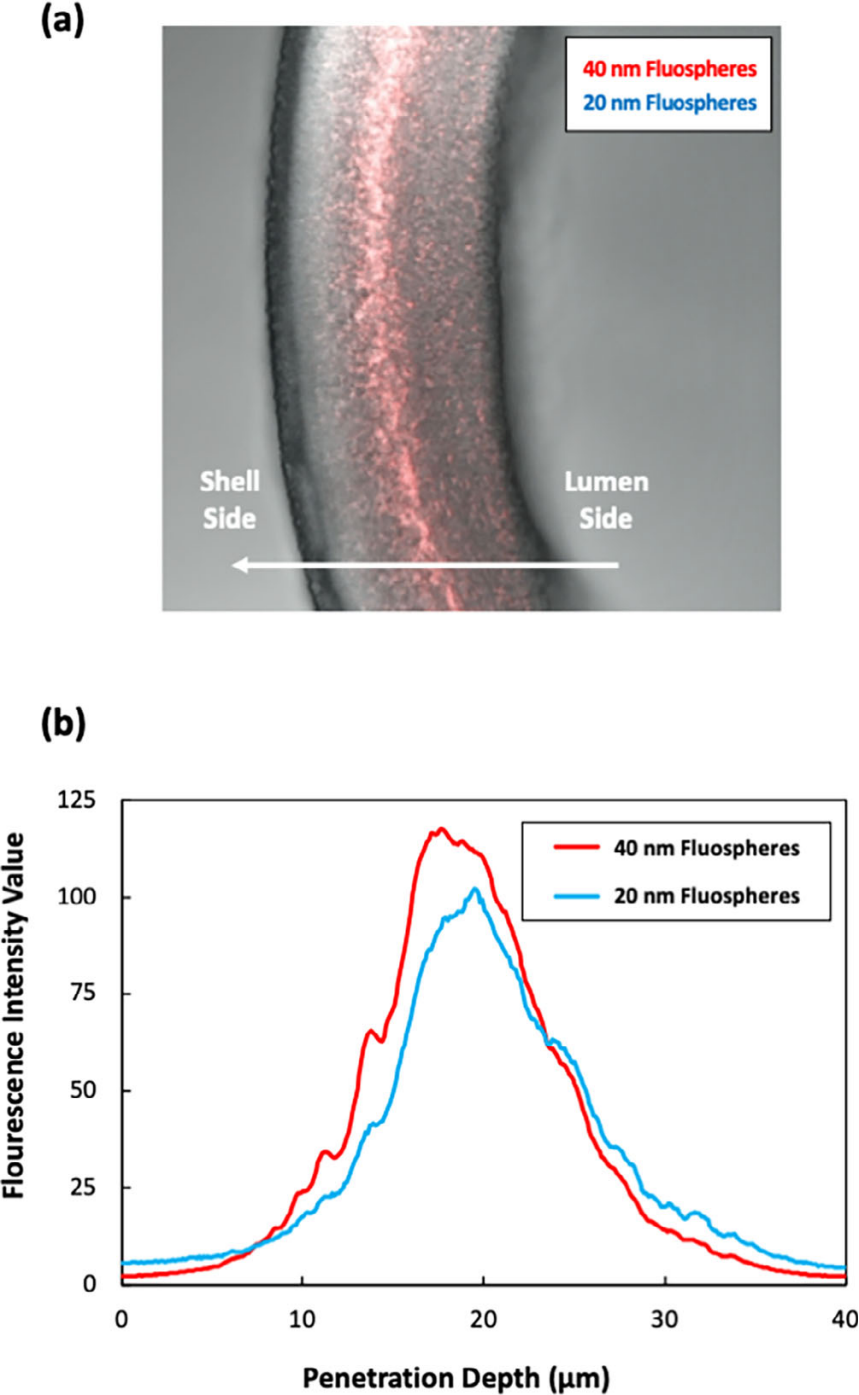
(a) Image of cross‐section of a single Planova™ 35N hollow fiber following constant‐flux filtration at 185 L/m^2^/h using 20 and 40 nm Fluospheres. Direction of filtration occurs from lumen side into shell side as depicted. (b) Fluorescence intensity vs. penetration depth profiles obtained from the fluorescent image.

Further insights into nanoparticle retention by the Planova 35 N filters were obtained from cross‐sectional imaging of fouled filters that were challenged with 50 L/m^2^ of a mixture containing ~6 × 10^12^ particles/mL of the 20 nm Fluospheres (blue) and 5 × 10^11^ particles/mL of 40 nm Fluospheres (red) at a constant filtrate flux of 185 L/m^2^/h. The addition of these larger 40 nm Fluospheres ensured a strong fluorescent signal of captured particles within the membrane for confocal imaging. The fluorescence signals from both Fluospheres are overlaid on top of a brightfield image of a single hollow fiber in Figure 3a. Both populations of Fluospheres are captured within a bright band that appears approximately midway along the depth of the ~40 μm thick hollow fiber membrane. Quantitative analysis of particle capture versus penetration depth was obtained using ImageJ image‐processing software, with the raw fluorescence intensity signal plotted as a function of the distance from the lumen surface in Figure 3b. The particle band spans the distance from about 10–30 μm, suggesting that the retained particles are present in a “reservoir zone” that is ~20 μm thick. Similar results have been reported previously for retention of 20 nm virus, bacteriophage, and fluorescent nanoparticles in commercially‐available parvovirus filters.^21^, ^22^, ^32^ Interestingly, there was no statistical difference in the capture profiles for the 20 and 40 nm nanoparticles, likely due to the significant overlap in nanoparticle size distributions (shown in Figure S1). However, separate experiments performed with significantly larger 100 nm Fluospheres showed capture right at the filter entrance, that is, at the lumen surface of the hollow fiber (Figure S2).

Based on the results in Figure 3, the virus transmission data in Figure 2 were analyzed using the internal polarization model originally presented by Jackson et al.^21^ A simple mass balance on the mobile particles in the “reservoir zone” within the virus removal filter is given as:

(3) $$V_{R}\frac{\mathrm{dC}}{\mathrm{dt}}=xJ_{v}C_{\text{feed}}-J_{v}S_{a}C$$

where C and $C_{\text{feed}}$ are the concentrations of mobile particles in the reservoir zone and feed, respectively. $V_{R}$ is the volume per unit area of the reservoir zone, $J_{v}$ is the (constant) filtrate flux, and x is the fraction of particles entering the reservoir zone that remain mobile (i.e., the fraction that is not irreversibly captured). $S_{a}$ is the actual sieving coefficient for the retentive region of the membrane, defined as the ratio of the particle concentration in the filtrate divided by that in the reservoir zone. Equation (3) is integrated to give:

(4) $$C=\frac{xC_{\text{feed}}}{S_{a}}+\left(C_{o}-\frac{xC_{\text{feed}}}{S_{a}}\right)e^{-\frac{S_{a}V}{V_{R}}}$$

where $C_{o}$ is the initial concentration of particles within the reservoir zone (typically equal to zero). The instantaneous virus/particle concentration in the filtrate is simply equal to $S_{a}C$.

The solid curve in Figure 2 is given by Equation (4) with the best‐fit values of x and $S_{a}$ determined using the nonlinear fitting function in Wolfram Mathematica giving x=0.89 and $S_{a}=9.0\times 10^{-4}$. The reservoir volume was evaluated as $V_{R}$ = 0.01 L/m^2^ (10 μm) based on the results in Figure 3, assuming a void fraction within the filter of 50%. The model is in excellent agreement with the data, properly capturing the increase in transmission with volumetric throughput as the AAV/Fluospheres accumulate in the reservoir zone. At large throughput, the exponential term in Equation (4) goes to zero, with the transmission approaching $\frac{C_{\text{filtrate}}}{C_{\text{feed}}}=x$, again in good agreement with the experimental results.

### 3.3 Effect of filtrate flux

**FIGURE 4:**
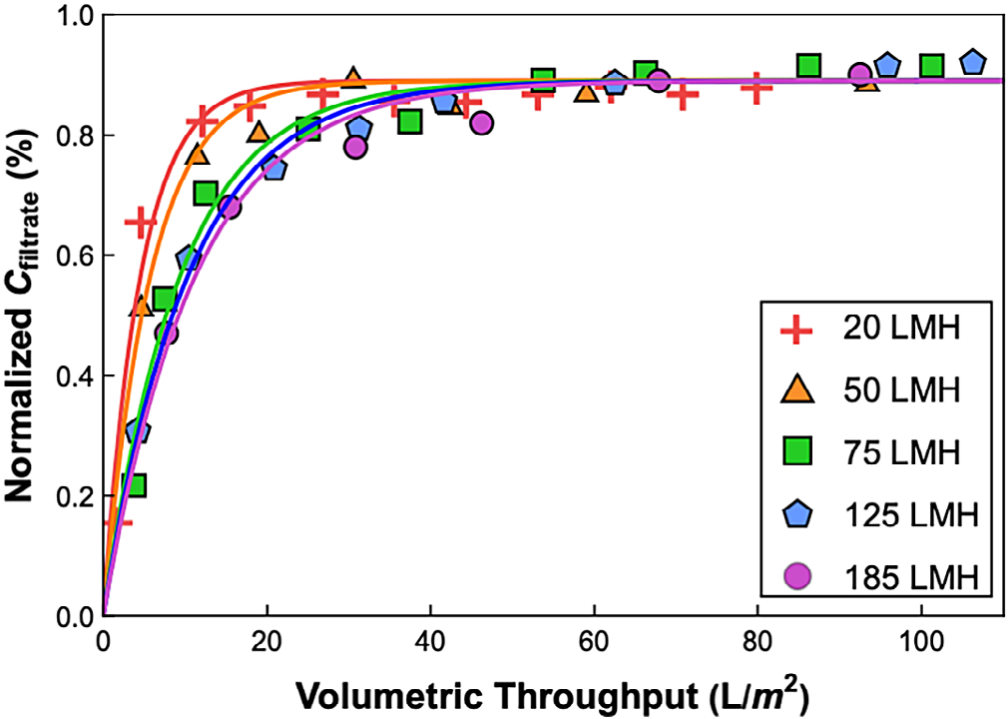
Normalized filtrate concentration vs. volumetric throughput for filtration of the 20 nm Fluospheres through the Planova 35N at filtrate flux from 20 to 185 L/m^2^/h. All experiments were performed at a feed concentration of ~7 × 10^11^ particles/mL. Solid curves are model calculations using Equation (4), with x=0.89 and $S_{a}$ varied across fluxes.

The effect of filtrate flux on the transmission of the 20 nm Fluospheres is shown in Figure 4. At all fluxes, the transmission increases with increasing volumetric throughput, similar to the data in Figure 2, with the asymptotic transmission essentially independent of the filtrate flux. However, the rate of increase in the transmission is fastest for the run at the lowest filtrate flux, even though the data are plotted as a function of the volumetric throughput (and not the filtration time), with the rate becoming progressively slower with increasing filtrate flux.

**FIGURE 5:**
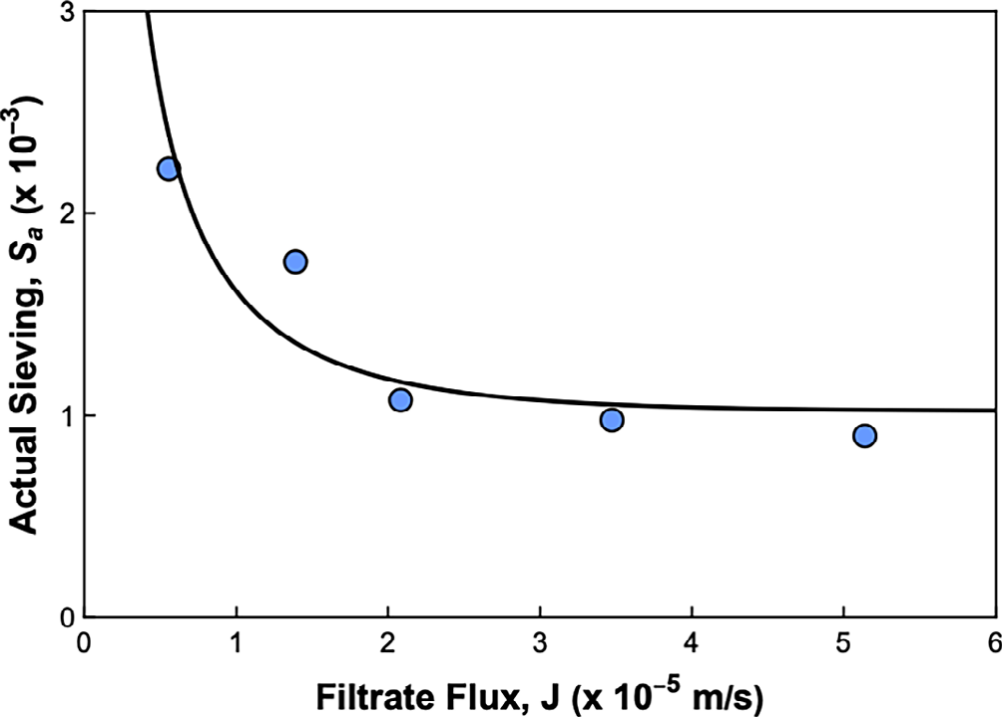
Actual sieving coefficient $S_{a}$ as a function of filtrate flux. Solid curves are model calculations using Equation (5), with fitted parameters $S_{\infty }=0.001$ and $\phi K_{d}=7.6\times 10^{-5}$. The model is in good quantitative agreement with the data, with $R^{2}=0.97$.

The solid curves in Figure 4 are again given by the internal polarization model using the same value of x=0.89 for all conditions but with the best fit values of $S_{a}$ (shown in Figure 5) determined separately at each flux. The actual particle sieving coefficient decreases from $S_{a}=2.2\times 10^{-3}$ at $J_{v}$ = 20 L/m^2^/h (5.6 μm/s) to $9.0\times 10^{-4}$ at $J_{v}$ = 185 L/m^2^/h (51 μm/s). This decrease in $S_{a}$ with increasing flux is consistent with predictions of available hydrodynamic models,^33^, ^34^ which predict a decrease in sieving coefficient due to the reduction in the contribution of particle diffusion to the overall rate of particle transport through the membrane at higher convective flow velocities.

The solid curve in Figure 5 is given by^33^, ^34^:

(5) $$S_{a}=\frac{C_{\text{filtrate}}}{C}=\frac{S_{\infty }e^{\mathrm{Pe}}}{S_{\infty }+e^{\mathrm{Pe}}-1}$$

where $S_{\infty }$ is the asymptotic value of $S_{a}$ at very large filtrate flux, that is, in the limit of convective‐dominated transport, and Pe is the membrane Péclet number, which describes the relative contribution of diffusive to convective transport through the membrane:

(6) $$\mathrm{Pe}=\frac{S_{\infty }}{\phi K_{d}}\left(\frac{J_{v}l}{D_{\infty }}\right)$$

l is the length of the selective “capillary” pores within the membrane, which has been estimated as ~120 nm for Planova filters.^35^ $D_{\infty }=1.6\times 10^{-11}\;m^{2}/s$ is the diffusion coefficient of particles in the bulk solution (determined from the DLS data), while $\phi K_{d}$ is the product of the partition coefficient ϕ and the hindrance factor for diffusion $K_{d}$. The data for $S_{a}$ are well described using the best‐fit values of $S_{\infty }=0.001$ and $\phi K_{d}=7.6\times 10^{-5}$ with $R^{2}=0.97$.

The effects of filtrate flux on the yield of AAV and the clearance of XMuLV are presented in Table 2. Data were obtained using AAV at a concentration between 10^10^ and 10^12^ vg/mL, spiked with ~5 log TCID_50_/mL of XMuLV (aside from the first run at 11.9 psi, which was performed using pure AAV). There was negligible fouling in these experiments, so the measured pressure and flux were essentially constant over the course of the filtration (less than an 8% flux decay was observed). AAV yield was >90% except for the single run at 185 L/m^2^/h, which provided a yield of only 87%. The lower AAV yield in this case could be the result of the higher loading of AAV onto the membrane and/or the higher filtrate flux. The average yield was slightly higher at lower fluxes, consistent with the results in Figure 5, although this effect was not statistically significant given the variability associated with the qPCR assay. Each row in Table 2 corresponds to a single experiment, with the error bars corresponding to the standard error associated with triplicate measurements of AAV yield. There was no measurable XMuLV in the filtrate samples under all three conditions, corresponding to LRV ≥ 4.3 even during operation at a relatively low flux of 45 L/m^2^/h.

**TABLE 2:** Yield of AAV and log‐reduction value of XMuLV at different operating pressures/filtrate fluxes. All data were collected using the Planova™ 35N filter.

Operating pressure (psi)	Filtrate flux (L/m^2^/h)	Buffer	AAV feed titer (vg/mL)	Volumetric throughput (L/m^2^)	AAV yield (%)	XMuLV LRV
11.9	185	PBS	7.2 × 10^11^	160	87% ± 1%	—
11.4	180	Tris	4.1 × 10^10^	40	98% ± 3%	≥4.9
7.0	115	Tris	4.6 × 10^10^	36	98% ± 9%	≥5.0
7.0	115	PBS	5.9 × 10^10^	37	97% ± 1%	≥4.3
2.8	45	Tris	4.1 × 10^10^	38	94% ± 3%	≥5.1

### 3.4 Process disruptions

**FIGURE 6:**
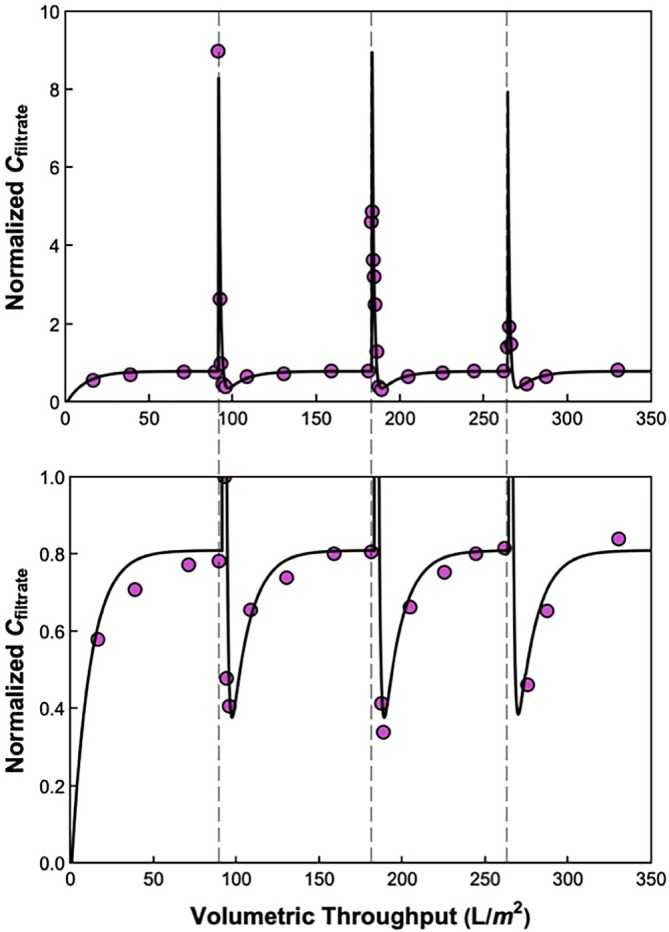
(a) Normalized filtrate concentration (C_filtrate_/C_feed_) as a function of volumetric throughput during operation at 185 L/m^2^/h. Process disruptions (denoted with the dashed lines) occurred as follows: Process pause at 91 L/m^2^, drop in flux to 30 L/m^2^/h at 182 L/m^2^, and drop in flux to 120 L/m^2^/h at 264 L/m^2^. (b) The local minimum in filtrate concentration after each disruption is made visible in the expanded view. Curves are model calculations using Equation (9), with x=0.81, y=0.07, $S_{m}=9.0*10^{-3}$, and $S_{a}$ varied based on the filtrate flux.

Several previous studies have demonstrated that a process disruption, for example, a shutdown or reduction in flux/pressure during the filtration process, can lead to significant virus breakthrough during parvovirus filtration. Figure 6 shows results for transmission of the 20 nm Fluospheres (at a feed concentration of ~7 × 10^11^ particles/mL) through the Planova 35N in response to several process disruptions (each denoted by a vertical dashed line). The filtration was started at a filtrate flux of 185 L/m^2^/h (transmembrane pressure of ~12 psi) for the first 91 L/m^2^, at which point the filtration was paused for 5 min (with the transmembrane pressure reduced to 0 psi). The nanoparticle transmission during the constant flux filtration stabilized at 81%, very similar to the results in Figure 2; the slightly smaller transmission is due to the use of a different batch of Fluospheres with slightly larger mean diameter. Immediately after the pause, the nanoparticle concentration in the filtrate increased by nearly a factor of 10, with the concentration in the first filtrate sample obtained after restarting the filtration being nine times larger than the feed concentration. The filtrate concentration then decreased rapidly, passing through a minimum of C_filtrate_/C_feed_ = 0.4 before increasing back to the same asymptote that was obtained before the disruption (made visible in the expanded view shown in the bottom panel of Figure 6). This clearly demonstrates that there was no damage to the filter due to the process disruption.

The filtration was continued at 185 L/m^2^/h until a volumetric throughput of 182 L/m^2^, at which point the flux was rapidly reduced to 30 L/m^2^/h (pressure of ~2 psi) for 5 min before being restored to 185 L/m^2^/h. A third disruption was performed at a volumetric throughput of 264 L/m^2^, with the flux reduced from 185 to 120 L/m^2^/h (pressure of ~8 psi). In each case, there was a rapid spike in the transmission in the first grab sample obtained after the disruption, with the nanoparticle concentration then going through a weak minimum before returning to the asymptotic value of ~81%. The magnitude of the “spike” in nanoparticle concentration was greatest for the disruption with zero flux and smallest for the disruption in which the flux was only decreased to 120 L/m^2^/h. This behavior is consistent with previous studies of process disruptions during parvovirus filtration, with the spike in virus transmission arising from the diffusion (release) of previously captured virus from within the reservoir zone, although it is important to note that experiments with parvovirus filters have never shown filtrate concentrations in grab samples that are nearly 10‐fold larger than the feed concentration.

The unusual concentration profiles seen in Figure 6 were described using a modified version of the process disruption model originally developed by Woods and Zydney.^22^ In this case, we assume that some fraction (y) of previously‐trapped particles are released during the process disruption, with these particles washed through the membrane during the subsequent filtration:

(7) $$C_{r}=\left(\frac{{\mathrm{yC}}_{\text{Feed}}\left(1-x\right)V^{*}}{V_{R}}\right)e^{\left(\frac{-S_{m}\left(V-V^{*}\right)}{V_{R}}\right)}$$

where $V^{*}$ is the volumetric throughput at which the disruption occurred. Note that Woods and Zydney also accounted for virus recapture following the process disruption (using fitted parameter “z”); this phenomenon did not appear to happen with this larger pore size filter (the best fit values of z were all approximately zero based on the data in Figure 6). The sieving coefficient that describes the washout of these released particles ($S_{m}$) was allowed to be different than the sieving coefficient for the particles in the original challenge since the particles can diffuse deeper into the filter during the process disruption. Similarly, it was assumed that the particles that had accumulated in the reservoir zone ($C_{m}$) during the disruption were also washed through the filter with a sieving coefficient $S_{m}$ when the filtration is restarted:

(8) $$C_{m}=\frac{xC_{\text{feed}}}{S_{a}}\left(1-e^{-\frac{S_{a}\left(V^{*}\right)}{V_{R}}}\right)e^{-\frac{S_{m}\left(V-V^{*}\right)}{V_{R}}}$$

The concentration of particles in the filtrate following each process disruption is thus given as the sum of three populations of particles—the “new” particles entering the filter after the process disruption, the mobile particles in the reservoir zone that accumulated during the initial filtration, and the previously captured particles that were released by the process disruption:

(9) $$C_{\text{filtrate}}=S_{a}C_{\mathrm{new}}+S_{m}\left(C_{m}+C_{r}\right)$$

where the concentrations in Equation (9) are the concentrations of the three particle populations in the reservoir zone at any instant in time.

The solid curve in the upper and lower panels of Figure 6 is the model calculation given by Equation (9), solved as a piecewise function over the multiple phases of the filtration. The best‐fit values of the model parameters were: x=0.81, y=0.07, and $S_{m}=9.0\times 10^{-3}$, with $S_{a}$ values as given in Figure 5. As mentioned previously, the slightly smaller value of x compared to the model plots in Figures 2 and 4 is due to the use of a different batch of Fluospheres with a somewhat larger diameter. The best fit value of y=0.07 suggests that 7% of the previously “captured” particles are released upon disruption. The best‐fit value of the sieving coefficient for the washout of these released particles ($S_{m}$) was 10× the value used to describe the behavior during the initial particle challenge, which reflects the diffusion of particles deeper within the filter during the process disruption. The model is in good agreement with the data, effectively describing both the large increase in the particle concentration in the filtrate samples obtained immediately after the process disruptions as well as the transient reduction in particle concentration arising from the washout of particles that had accumulated in the reservoir zone. The very large concentrations in the first grab samples after the process disruption are a direct result of the large value of $S_{m}$ which leads to the rapid transmission of particles that were previously accumulated in the reservoir zone during the first stage of the filtration.

**FIGURE 7:**
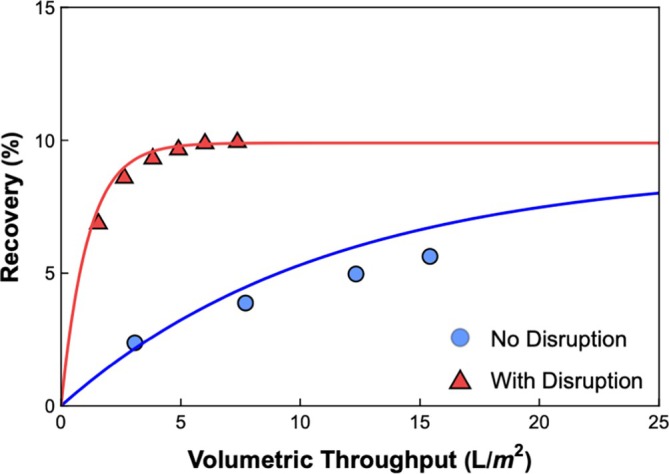
Nanoparticle recovery during buffer flushing procedures both with (in red) and without (in blue) a 30 min process disruption at the end of the run. Virus filtration was performed at a constant filtrate flux of 185 L/m^2^/h with 7 × 10^11^ nanoparticles/mL.

The results in Figure 6 suggest that it should be possible to use a process disruption to increase the overall yield of AAV during the virus removal filtration step. This effect was explored by performing a typical particle challenge at a constant filtrate flux of 185 L/m^2^/h followed by a buffer chase, both with and without a 30 min process disruption immediately before the buffer chase. The Fluosphere recovery during the 100 L/m^2^ filtration was ~80%; the 20% loss of particles is consistent with the results presented previously. Figure 7 shows the Fluosphere recovery during the buffer chase. When the buffer chase is initiated immediately after the filtration, without any process disruption, only 6% of the Fluospheres were recovered after 15 L/m^2^ of buffer. In contrast, ~10% of the Fluospheres were recovered after only 5 L/m^2^ of buffer when the buffer chase was implemented after a 30 min disruption. This gave a total Fluosphere recovery of 90%, with only a 5% dilution, compared to 86% recovery with 15% dilution when the buffer chase was implemented without any process disruption.

The solid curves in Figure 7 correspond to the model calculations, with nanoparticle recovery evaluated by integrating the normalized filtrate concentration profiles with respect to volumetric throughput. The decay in filtrate concentration during the buffer chase was evaluated by integrating the mass balance without the input term as described by Afzal and Zydney.^16^ The model is in excellent agreement with the data using the values of the model parameters determined from the data in Figure 6; none of the parameters were fit to the data in Figure 7. The model predicts that a standard buffer chase (without a process pause) can only recover 9% of the particles even at very large buffer flush volumes; these are the mobile particles that were accumulated in the reservoir zone during the initial filtration. For comparison, the particle recovery after the process disruption is increased to slightly more than 10% due to the recovery of some of the previously captured particles during the initial challenge. The model equations can thus provide a framework that can be used to optimize the buffer chase accounting for both AAV recovery and dilution.

The impact of process disruptions was also examined using AAV (at a feed concentration of ~2 × 10^10^ vg/mL) spiked with 4–5 logs TCID_50_/mL of XMuLV. Experiments were carried out at operating pressures of 2.8, 7.0, and 11.4 psi, with one or two 30 min process disruption(s) (with zero pressure during the disruption), followed by a buffer chase of 10 L/m^2^ at the same pressure. Results are summarized in Table 3. In each case, the AAV yield was >99%, which is a statistically significant improvement over the results presented in Table 2, confirming that the incorporation of process disruptions can be used to improve AAV yield. Importantly, the process disruptions had no statistical impact on the LRV of the larger virus. The LRV of XMuLV remained greater than 5.0, with no breakthrough detected in the TCID_50_ assay.

**TABLE 3:** Yield of AAV and log‐reduction value of XMuLV during filtration through the Planova 35N at varied operating pressures following 30 min process disruption(s).

Operating pressure (psi)	Filtrate flux (L/m^2^/h)	Number of 30‐min pauses	AAV yield (%)	XMuLV LRV
11.4	180	2	>99%	≥ 5.5
7.0	115	1	>99%	—
2.8	45	2	>99%	≥ 5.0

## 4 CONCLUSIONS

This study represents the first detailed investigation into the effects of filtrate flux and process disruptions on virus filtration performance with AAV therapeutics. Purified AAV capsids and carboxylate‐modified polymeric nanoparticles with a nominal diameter of 20 nm were used as feed material—these specific nanoparticles were chosen given their similar Z‐average size and zeta potential as AAV. Virus filtration experiments confirmed that the model nanoparticles act as an effective surrogate for the AAV, with both systems exhibiting nearly identical transmission profiles. AAV and nanoparticle filtration performed at various filtrate fluxes (ranging between 20 and 185 L/m^2^/h) suggest that moderately higher AAV yield may be obtained during operation at lower fluxes, consistent with the effects of diffusion on particle transmission. High removal of xenotropic murine leukemia virus (LRV ≥ 4.3) was obtained even during operation at fluxes as low as 45 L/m^2^/h.

AAV and nanoparticle transmission/recovery were also assessed following several different process disruptions. The instantaneous drop in flux during the disruption facilitated the recovery of previously‐trapped particles, with greater recovery observed during a process pause compared to that when the flux was simply reduced during the disruption. The filtrate concentration in a small grab sample taken immediately after a 5 min process pause was 9× higher than the concentration just before the disruption due to the release of previously captured particles in combination with the enhanced washout of particles that had accumulated in the reservoir zone. These results suggested that process pauses may be used advantageously within AAV manufacturing to improve overall step‐yield. This was verified by performing typical filtration followed by a buffer chase, both with and without a 30 min process pause just before the buffer chase. A total nanoparticle recovery of 90% with only a 5% dilution was observed when the process pause was incorporated, compared to 86% recovery with 15% dilution when the buffer chase was implemented without any process disruption. Experiments performed with real AAV systems confirmed that such process disruptions may be used to obtain as high as 99% AAV yield while maintaining very high clearance of XMuLV (LRV ≥ 5). Future studies will be needed to examine the impact of process disruptions on the clearance of other (smaller) model viruses like SV40 to confirm the robustness of this approach to achieve high AAV yield while maintaining the required level of virus removal.

The particle transmission behavior was well described using a modified internal polarization model accounting for particle capture and particle accumulation within a “reservoir zone” which was visualized by confocal microscopy. The actual sieving coefficient of particles through the reservoir zone was found to decrease with increasing filtrate flux, in good agreement with previous results for parvovirus filters^16^, ^17^ and with predictions of available hydrodynamic models accounting for the relative contributions of particle diffusion and convection (as described by the membrane Péclet number). Interestingly, the internal polarization model suggests that the particle‐sieving coefficient was increased by 10‐fold following a process disruption, an effect that has not been previously identified in studies with parvovirus retentive filters. This increase in particle sieving coefficient is likely due to the diffusion of particles deeper through the membrane during the process disruption. The combined data and modeling offer a practical framework to guide the design of virus removal filtration processes that maximize both AAV yield and viral clearance.

## AUTHOR CONTRIBUTIONS

**Akshay S. Chaubal:** Data curation (lead); formal analysis (lead); investigation; writing—original draft. **Asingsa W. Arachchige:** Data curation; formal analysis; investigation; writing—review and editing. **Annabelle J. Zahn:** Data curation; investigation; writing—review and editing. **S. Ranil Wickramasinghe:** Conceptualization; funding acquisition; supervision; writing—review and editing. **Xianghong Qian:** Conceptualization; funding acquisition; supervision; writing—review and editing. **Andrew L. Zydney:** Conceptualization; funding acquisition; investigation; methodology; supervision; writing—review and editing.

## CONFLICT OF INTEREST STATEMENT

The authors declare no conflict of interest.

## Supporting information


**Figure S1:** DLS size distributions (by intensity) for Fluospheres with nominal diameters of 20, 40, and 100 nm. Note the significant overlap between the size distributions of the 20 and 40 nm nanoparticles, thereby explaining the similar confocal capture profiles between those two systems (as presented in Figure 3 in the text).
**Figure S2:** Image of cross‐section of a single Planova™ 35 N hollow fiber following constant‐flux filtration at 185 L/m^2^/h using 100 nm Fluospheres. Direction of filtration occurs from lumen side into shell side as depicted. The large particles are captured right at the filter entrance, that is, at the lumen surface of the hollow fiber.

## Data Availability

The data that support the findings of this study are available from the corresponding author upon reasonable request.
