# Supplementary material for: Performance of large virus removal filters during AAV processing: Influence of flux and process disruptions
Source: Biotechnol Prog. 2026 Jan 16;42(2):e70106. doi: 10.1002/btpr.70106 (PMC13055148; doi:10.1002/btpr.70106)
Supplement: Supplementary file 1 — Figure S1: DLS size distributions (by intensity) for Fluospheres with nominal diameters of 20, 40, and 100 nm. Note the significant overlap between the size distributions of the 20 and 40 nm nanoparticles, thereby explaining the similar confocal capture profiles between those two systems (as presented in Figure 3 in the text). Figure S2: Image of cross‐section of a single Planova™ 35 N hollow fiber following constant‐flux filtration at 185 L/m2/h using 100 nm Fluospheres. Direction of filtration occurs from lumen side into shell side as depicted. The large particles are captured right at the filter entrance, that is, at the lumen surface of the hollow fiber. [file BTPR-42-e70106-s001.docx]

**Supplementary Information**

**Figure S1:** DLS size distributions (by intensity) for Fluospheres with nominal diameters of 20, 40, and 100 nm. Note the significant overlap between the size distributions of the 20 and 40 nm nanoparticles, thereby explaining the similar confocal capture profiles between those two systems (as presented in Figure 3 in the text).

**Figure S2:** Image of cross-section of a single Planova^TM^ 35N hollow fiber following constant-flux filtration at 185 L/m^2^/h using 100 nm Fluospheres. Direction of filtration occurs from lumen side into shell side as depicted. The large particles are captured right at the filter entrance, i.e., at the lumen surface of the hollow fiber.
